# Report of a Case of Ovariotomy

**Published:** 1884-01

**Authors:** W. H. Kane King

**Affiliations:** Mt. Sterling, Ill.


### ARTICLE IV.

### REPORT OF A CASE OF OVARIOTOMY. By W. H. KANE KING, M.D., Mt. Sterling, Ill.

On Monday, October 8th, I was called in consultation with Dr. G. H. Tebo, to examine Miss W., aged 23, living four miles in the country, the doctor diagnosticating ovarian disease. The patient had first observed enlargement one year before, but now the tumor reached the middle of the third stage (Peaslee). Fluctuation being marked, the aspirator withdrew eight liters (seven quarts) of thin, straw colored fluid, after which the polycystic nature of the tumor was apparent. Fluid straw color; alkaline in reaction; sp. gr. 1018; no albumen, paralbumen, or metalbumen in fluid from the main cyst. The fluid of the other cysts was not tested. On boiling with nitric acid an exceedingly fine, dense foam rose high above the fluid, and remained there after cooling, a circumstance I have not before observed in testing fluids from the cervix. The following Monday was set for the operation, and Dr. Tebo kindly asked me to perform it We placed the patient on a fluid diet; on Friday night gave calomel and soda; on Sunday morning, oil; and Sunday night a dose of codia. On Monday morning, quinia gr. x., with nothing to eat after 8 o'clock. My home assistant, Dr. Lizzie White, had carefully prepared sponges, ligatures, and finger cloths, boiling them in a carbolated solution, and at 1 o'clock the instruments were lying in a warm solution of the same disinfectant. A tub of warm salt water stood under the table. The patient was etherized, and placed on the table by Dr. Owen Fulton. The room had been whitewashed, divested of furniture, and the floor sprinkled with carbolized water. Assisted by the above and Drs. Baxter and Mears, I proceeded thus: An incision in the usual place below the umbilicus, four and a half inches long, exposed the refilled tumor. All oozing was stopped, and not one drop allowed to reach the peritoneal cavity. The main and some minor cysts were then emptied with trocar and tube. The point of puncture was tied. No adhesions found. A very short, wide pedicle involved the whole left ligamentum alaris. I transfixed it with a needle armed with double ligature, locked and tied it in halves; another strong ligature was passed around the base of the tumor, and the pedicle was surrounded with a finger cloth, and severed with scissors. Then I frosted the cut end of the pedicle with a warm iron, and left it, not poerated, but only a half inch long, standing at right angles with the uterus and touching nothing. So far scarcely one drop of blood had contaminated the parts. Yet a sponge wrung from a solution of sodium was passed to the most dependent part. A sponge on the end of a catheter was now placed in Douglas' cul-de-sac, and a piece of carbolized flannel placed over the bowels. The external opening was closed by four silver and seven silk sutures. The former were thus arranged: A needle armed with a "carrier thread" carried the wire from the peritoneal side (always) one inch from the cut edge to a point on the skin one inch from the cut. In this way the four wires were first placed on one side only. Then a carbolized pine slat, with rounded corners and ends, of suitable length, was perforated with an awl, to receive the ends of the wire. A large glass bead was placed on each, and they were then tied, the two upper together and the two lower. Then the other ends were carried in a similar way through the opposite side. The flannel and sponge being now removed clean, the perforated pine, quill, and the beads were adjusted, and the wound closed, great care being observed to coapt the peritoneal surfaces throughout the extent of the wound. Seven light silk sutures closed the wound *perfectly*. The usual carbolized (oil) dressings were applied, and the patient "hot bottled" in bed. In one hour she awoke, as if from gentle sleep, and evinced none of the shock usually observed. The time occupied was one hour and a quarter, which was mostly consumed in means directed to prevent the fluid or blood from entering the abdominal cavity, mopping, etc.—time, as I believe well spent. The fifth day, union being perfect, the silk sutures were removed, and on the ninth day the silver ones, not one drop of blood, pus, or oozing could be discovered. The temperature averaged about 99 7/10 102 it reachedwice ½. Quinia in large t ; doses was then given ; at other times in small doses. She vomited once on the second and third days. The concavity of the abdominal surface at any time would have held a cup of water (due to preparatory treatment). Dr. Tebo informs me to-day that she is doing well, and is able to sit up. This is the second case successful here in pure country air, and I doubt the propriety of operations of this kind in crowded cities and hospitals.

